# Is Dentistry a Specialty of Medicine?

**Published:** 1907-04

**Authors:** J. C. Richard

**Affiliations:** Milledgeville, Ga.


### IS DENTISTRY A SPECIALTY OF MEDICINE?

By J. C. Richard, Milledgeville, Ga.

With great pleasure and interest I have perused the contributions appearing in our dental journals pleading for a closer relationship between medicine and dentistry.

Both professions would be greatly benefited by such a step. Some in each profession are urging the recognition of dentistry as a specialty of medicine. There seems to be a marked indifference on the part of the medical profession to the proposed alliance, while dentistry stands like one of Dickens' characters. "Barkis is willing."

Why this indifference on the part of our professional bretheren? Is it because we are not understood or considered merely mechanics? Do they place the estimate on us today that was placed on us yesterday? Do they consider our work restricted and contracted as was formerly the case? Surely in this day of multiplied periodicals no professional man can entertain any such views of the scope of our work.

The progress of our profession has been marvelous, exceeding the limits of the most optimistic prophet. No profession can measure arms with us in this respect. In fact we have outstripped all professions in the rapid strides of development. Occasionally you find a physician discounting the ability of the dentist, but usually he is one of the "has beens." I am glad to state that generally physicians of professional superiority place the proper estimate on the ability of the dentist.

In finding it necessary to administer nitrous oxide gas to a female patient for the removal of teeth, the appointment was made. The patient was given the usual instructions as to emptying bladder, wearing loose clothing, etc. When the time for the operation came she was accompanied by her physician. He called me aside and said, "Doctor I know nothing of the administration of nitrous oxide gas. There was no necessity of my coming, but Miss W— insisted on it. What restoratives do you use?" I replied, "Amyl nitrite or sulphate of strychnine." The operation was performed and patient revived without having to resort to restoratives.

Now, that the patient did not suspect that her physician was entirely ignorant of nitrous oxide gas and the restoratives used, but on the contrary, had the utmost confidence in his knowledge, relying little on the dentist. The greatest barrier to our expansion is the lack of confidence in our ability on the part of our patients. How often when suggesting some mode of procedure, we are confronted with the remark: "My doctor does not think it best." It is within the power of the physician to correct this low estimate the public has of us, and he owes it to us to do so.

Is the gap between medicine and dentistry kept open because of the many unethical advertising parlor dentists? These smooth tricksters; these professional parasites, these unscrupulous fellows, who cut price, quality, skill and rectitude? These fellows who would prostitute the profession, who by their suicidal methods are unconsciously perhaps digging their own graves? I say is it on account of these professional outcasts that we are not in closer relationship with the physician? No. Side by side in our daily papers stand the advertisments of both professions, each with beguiling solicitations striving to draw the unsuspecting shopper into his net. Surely we are not measured by the standing of those to whom we refuse fellowship. As well condemn the patriot because of the traitor. We are not responsible for these fellows. Every flock has its black sheep, every army its deserter, every fraternal organization its interloper, every church its hypocrite. No, it is not because of the unethical practitioner that we are not accorded a closer relationship with the medical fraternity.

It has been claimed by some writer that we are as truly specialists of medicine as the oculist, rhinologist or aurist; that a professional man, devoting his practice to a certain organ, or group of organs, is a specialist of medicine. This is not as specific as should be, and of course it is implied that any one practicing a specialty or posing as a specialist in medicine is equipped to carry on intilligently and scientifically his chosen line of work. He must by study and clinics perfect himself before he can be truly recognized as master of his vocation.

Are we as dentists, mentally equipped and fitted sufficiently to be called specialists in medicine? Of course we are familiar with the fact that the neurologist and other specialists have obtained the degree of M. D. before they have taken up their respective specialties; and there is the great difference between the dentist and the medical specialist. We are not M. D.'s and our knowledge of the course of study followed by the M. D. is meagre indeed. How many of us are competent to ligate an artery? How many can set a fractured bone? How many are familiar with the treatment of the milder diseases of the human body? Who of us can make a thorough examination of the heart? Who can analyze saliva? I say ignorance on our part of these and other items constitutes the great separating breach between medicine and dentistry; and we cannot expect a very close relationship between the two professions until we become more familiar with at least the minor details of medicine.

Why, sometime ago a dentist injected morphine for the relief of blind abscess. The agent failing to give relief, and the patient suffering intensely, he called in a physician. The physician very quickly found the cause of failure of action of morphine that the capillaries were contracted, therby preventing the absorption of the agent. This was overcome by the administration of three ounces of whiskey. Now, is the dentist equipped or prepared to be recognized as a specialist of medicine? Doubtless he is not an exception to the rule and perhaps is a fair representative of the rank and file of the profession.

If we expect to be recognized as specialists of medicine we must become familiar with medicine and surgery. The question very naturally arises how can we attain this end. That is, we who are actively engaged in the practice of our professions.

1st. Subscribe for one or more medical journals.

2nd. Attend all medical clinics and post mortem examinations possible.

3rd. Associate more with physicians.

4th. Study persistently those branches necessary.

5th. Join the medical society in your community, even if an honorary member.

6th. Desist from calling in physicians in our practice. Let us meet the emergency ourselves.

